# Supplementary material for: Transcriptome profiling of maize transcription factor mutants to probe gene regulatory network predictions
Source: G3 (Bethesda). 2024 Nov 20;15(1):jkae274. doi: 10.1093/g3journal/jkae274 (PMC11979765; doi:10.1093/g3journal/jkae274)
Supplement: jkae274_Supplementary_Data [file jkae274_supplementary_data.zip › Figure_S2_G3-2024-405474.pdf]

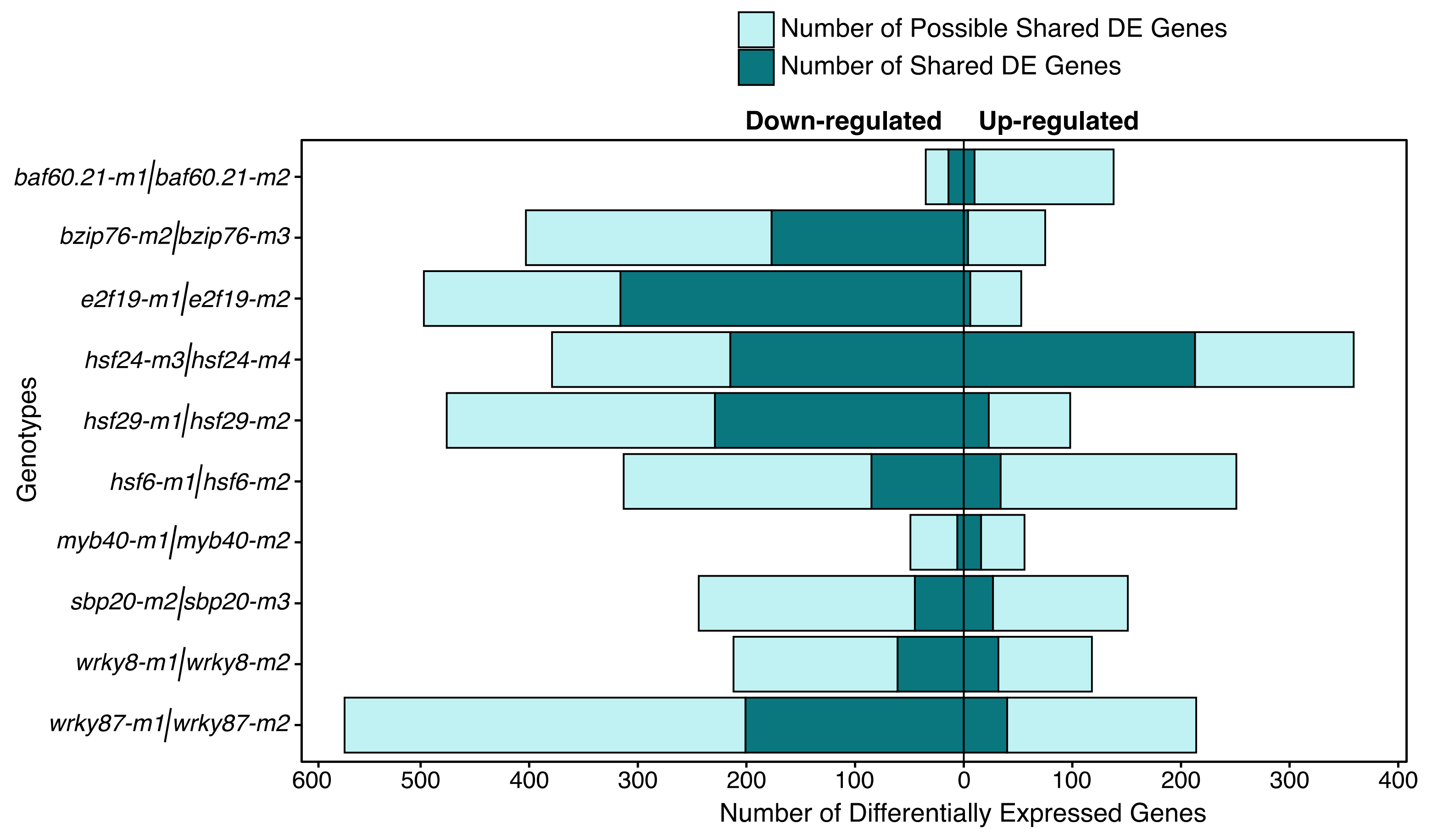

Number of Possible Shared DE Genes
Number of Shared DE Genes
Down-regulated
Up-regulated
Genotypes
baf60.21-m1|baf60.21-m2
bzip76-m2|bzip76-m3
e2f19-m1|e2f19-m2
hsf24-m3|hsf24-m4
hsf29-m1|hsf29-m2
hsf6-m1|hsf6-m2
myb40-m1|myb40-m2
sbp20-m2|sbp20-m3
wrky8-m1|wrky8-m2
wrky87-m1|wrky87-m2
600
500
400
300
200
100
0
100
200
300
400
Number of Differentially Expressed Genes
