# Supplementary material for: Transcriptome profiling of maize transcription factor mutants to probe gene regulatory network predictions
Source: G3 (Bethesda). 2024 Nov 20;15(1):jkae274. doi: 10.1093/g3journal/jkae274 (PMC11979765; doi:10.1093/g3journal/jkae274)
Supplement: jkae274_Supplementary_Data [file jkae274_supplementary_data.zip › Supplemental_Materials_Legends.docx]

**Supplemental Material Legends**

**Figure S1. Principal component analysis (PCA) of RNA-seq data.** PCA was performed separately on the RNA-seq samples for each of the 5 tissues: a) embryo imbibed, b) seedling leaf, c) coleoptile tip, d) tassel stem, e) tassel. Different symbols/colors are used for each genotype and ellipses are used to show the group of three biological replicates (except for *gras52-m1* which only has 2 replicates). Clustering was performed using CPM values for all genes that are expressed at > 1 CPM in control samples in the relevant tissue. Gene CPM values were arcsine transformed and scaled to have unit variance. The PCATools package in R was used to calculate principal components.

**Figure S2.** **Proportion of shared out of possible down- or up-regulated differentially expressed (DE) genes for TFs with multiple independent mutant alleles.** The number of shared DE genes (dark shading) as a proportion of the total possible DE genes that could be shared between alleles (light shading) for either up- or down-regulated DE gene sets. For all 10 TFs, the proportion of both down- and up-regulated DEGs shared between the two mutant independent alleles per TF (dark shading) represents a significant hypergeometric enrichment (p < 0.05) of finding more than the expected number of shared DEGs.

**Figure S3. GO-based analysis of TF mutant up-regulated differential expression.** A GO analysis of the up-regulated genes for all 32 mutants was used to identify a set of the top 50 non-redundant enriched terms (lowest p-values). The GO enrichment levels were determined by a hypergeometric test, where the GO term observed/expected number for each gene set was tested. Circles are used to indicate each significant enrichment of GO terms for 25 of the 32 mutants (*hsf20-m1, hsf29-m1, myb40-m1, myb40-m2, wky8-m1, wrky8-m2, wrky87-m2* have no significant enrichments in the top 50 GO terms for up-regulated DEGs).

**Figure S4. The number of predicted targets for each TF gene with mutant alleles.** Prior GRN work using yeast one-hybrid (Y1H) or gene co-expression networks (GCNs) have predicted putative targets for many maize TFs. For the 22 TFs assessed in this study the number of predicted targets based on a) Y1H or b) GCNs are shown for the mutant alleles tested. These values represent the number of predicted targets based on analysis of B73v4 gene annotations. The Y1H targets include genes identified in Yang *et al.* 2017 as well as additional targets based on further screening from Abnave *et al.* 2024. The GCN predicted targets identified in Zhou *et al.* 2020 were filtered to require syntenic ortholog annotations in W22 and expression of CPM > 1 in the tested tissue. Tan (n3) indicates predicted targets identified in at least 3/45 GCNs while brown (n1) indicates targets detected in at least one of the 45 GCNs.

**Figure S5. Expression changes for phenylpropanoid pathway genes in the TF mutants.** The relative expression in mutant compared to wild-type is shown for 61 phenylpropanoid pathway gene Y1H targets. The phenylpropanoid pathway genes are ordered by pathway branch (general, flavonoids, lignins) and enzyme with color coding on the right side of the plot. Y1H positive interactions are shown using an asterisk (*). The relative expression is indicated using color [DE Up or Down: differentially expressed (log_2_ fold change ≥ 1 and FDR adjusted p < 0.05); Trending Up or Down: fold change ≥ 1.5, but not differentially expressed; Not DE: not differentially expressed and fold change < 1.5; Untested: not expressed (CPM < 1) or no W22 gene annotated].

**Figure S6. Expression differences of three Y1H predicted target genes in TF regulator mutant and wild type genotypes.** The CPM values for each biological replicate (n ≥ 3) are for shown for three phenolic genes from Y1H TF-target predictions: *MYB40-A1, HSF24-Bz1, and E2F19-HCT11*. Mutant allele (pink) and wild-type W22 (blue) biological replicates are plotted comparatively with significant differential expression indicated (*); log_2_ fold change ≥ 1 and FDR adjusted p < 0.05.

**Figure S7. Enrichments of gene co-expression network (GCN) n3 predictions for some TF mutants.** The predicted targets for each mutant were identified based on co-expression interactions detected in at least 3 of the 45 GCNs (GCN n3) (File S2). a) For each mutant allele we calculated the fold-enrichment of DE genes that were predicted targets as the observed number of GCN n3 predicted target genes that were significantly DE divided by the expected number of DE predicted targets. Enrichments are only shown for the mutants that exhibit significant enrichment (*, p < 0.05) in at least 1/45 GCNs (n1) - see Figure 7a. b) The proportion of GCN predicted targets that are up- (red) or down-regulated (blue) in each mutant is shown. For each allele, significant hypergeometric enrichment for up- and/or down-regulated DE target genes were marked (*).

**Table S1. 32 Maize TF mutant alleles isolated from the UniformMu population to test GRN predictions.** Mutant allele transcriptome data obtained, UniformMu Mu ID and Stock, Pos: Mu insertion position within the gene, Pedi: pedigree of the mutant stock sampled for RNA-seq (BC = backcross, S = self), and PDI: protein-DNA interaction prediction method. RNA-seq CPM and FPKM for TF mutant and wild-type W22, DE data averaged across biological replicates (N), log_2_fc: log_2_ fold change of mutant to control, lfcSE: log fold change standard error and FDR adjusted p-value. Data for TF genes that are that are DE in the mutant compared to the wild-type are highlighted.

**File S1. Differentially expressed genes for each TF mutant allele.** Up- and down-regulated DE genes (log_2_fc ≥ 1 and FDR adjusted p-value < 0.05) for each mutant allele and those shared by multiple independent alleles per TF in this study.

**File S2. Morphological traits measured for a subset of TF mutant alleles.** Morphological traits were measured for 12 mutant alleles in two different fields during one field season. Measurements for two plant architecture traits (measured in cm): plant height (PHT) and ear height (EHT) and two flowering time dates: days to tassel shedding (DTT) and days to ears silking (DTE) are recorded for each mutant row and multiple W22 control rows per field (X-6 or X-7). Significance test (unpaired t-test, FDR adjusted p-value < 0.05) results for log transformed PHT and EHT values between each mutant row and the combined W22 control rows within a field are included.

**File S3. Metabolite data for 24 phenolic compounds in 32 TF mutant alleles.** The first sheet contains the raw LC-MS data arbitrary units of area (AUA) and limits of detection (LOD) for each phenolic compound per genotype, biological replicate and LC-MS batch (1 or 2). The mean AUA and standard deviation for the three biological replicates are included. AUA was log_2_ normalized for statistical analyses. The second sheet, log_2_ AUA t-test, contains the unpaired t-test statistic, raw p-values, and FDR adjusted p-values for each mutant allele compared to both controls: W22 r-g or UniformMu W22 (ufmu). Significance (FDR adjusted p-value < 0.05) is denoted by an asterisk (*) and not significant (ns).

**File S4. Significant hypergeometric enrichment of GO terms associated with TF mutant allele differentially expressed genes.** The subset (n) of TF mutant allele up- or down-regulated DEGs (B73v4 and W22 gene IDs recorded) with a significant GO term enrichment (hypergeometric, p-value < 0.05).

**File S5. Predicted targets from GCN and Y1H methods for all transcription factors in this study.** TF-target predictions are listed by prediction method: y1h; Y1H screen, n1; 1/45 GCNs, and/or n3; 3/45 GCNs. For TFs with Y1H predictions, total targets (n) represent all B73v4 target genes. For TFs with GCN predictions, total targets (n) represent a subset of the predicted B73v4 targets that have an annotated W22 gene model and are expressed (CPM ≥ 1 in at least one sample/tissue).
