# Supplementary figures and images for: Transcriptome profiling of maize transcription factor mutants to probe gene regulatory network predictions
Source: G3 (Bethesda). 2024 Nov 20;15(1):jkae274. doi: 10.1093/g3journal/jkae274 (PMC11979765; doi:10.1093/g3journal/jkae274)

**a** imbibed embryo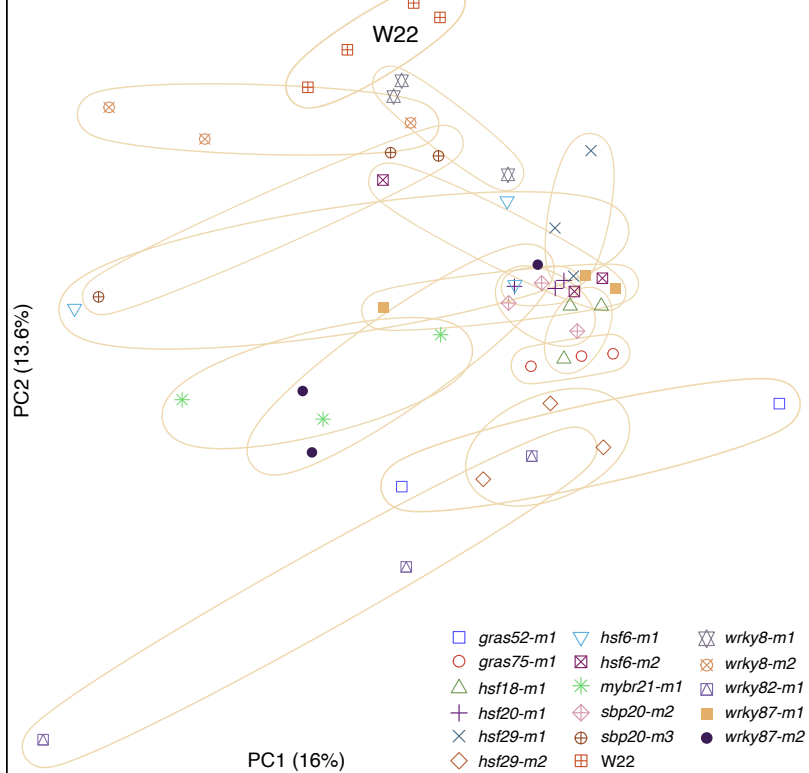**b** seedling leaf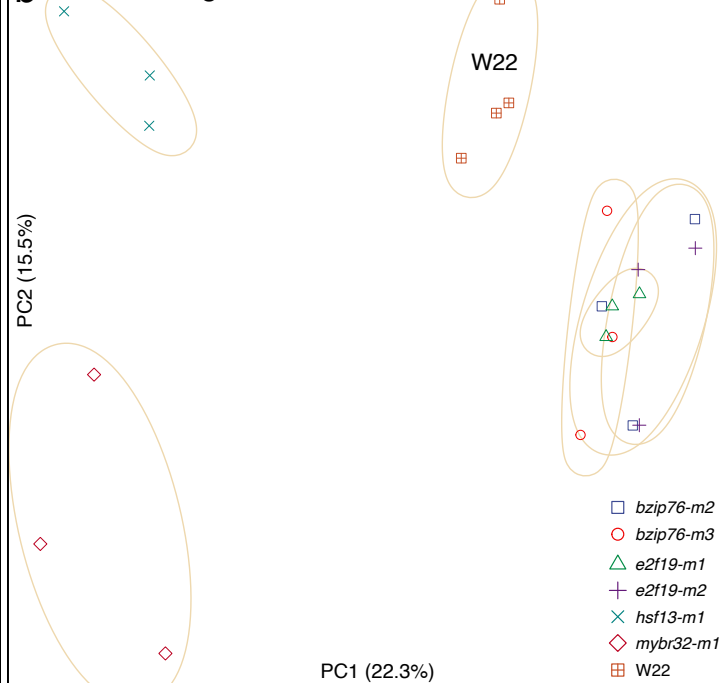**c** coleoptile tip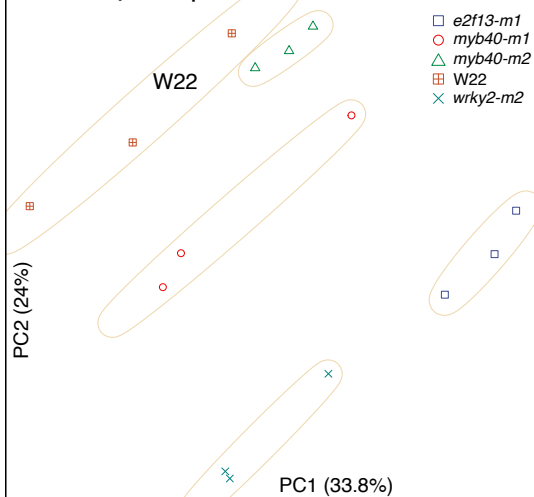**d** tassel stem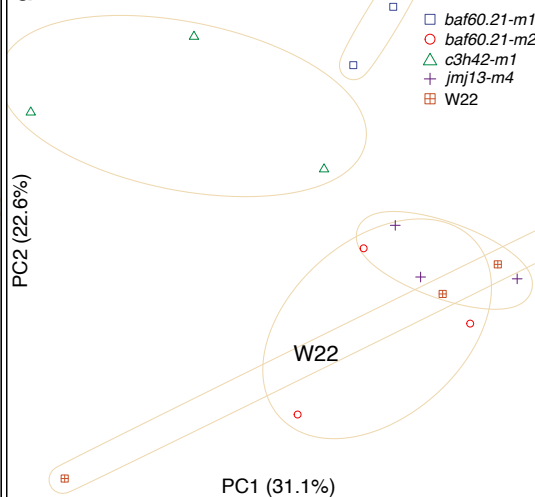**e** tassel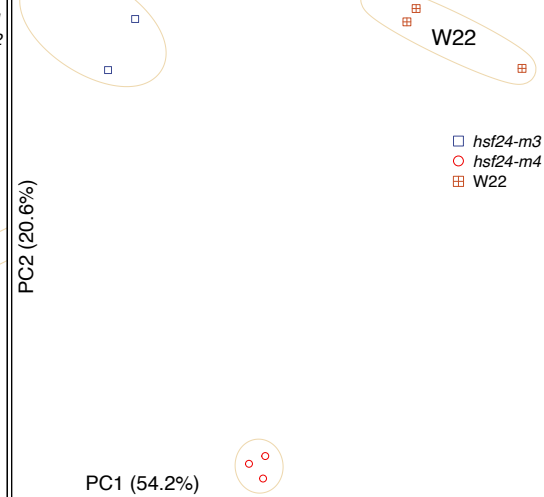

Supplement: jkae274_Supplementary_Data [file jkae274_supplementary_data.zip › Figure_S1_G3-2024-405474.pdf]

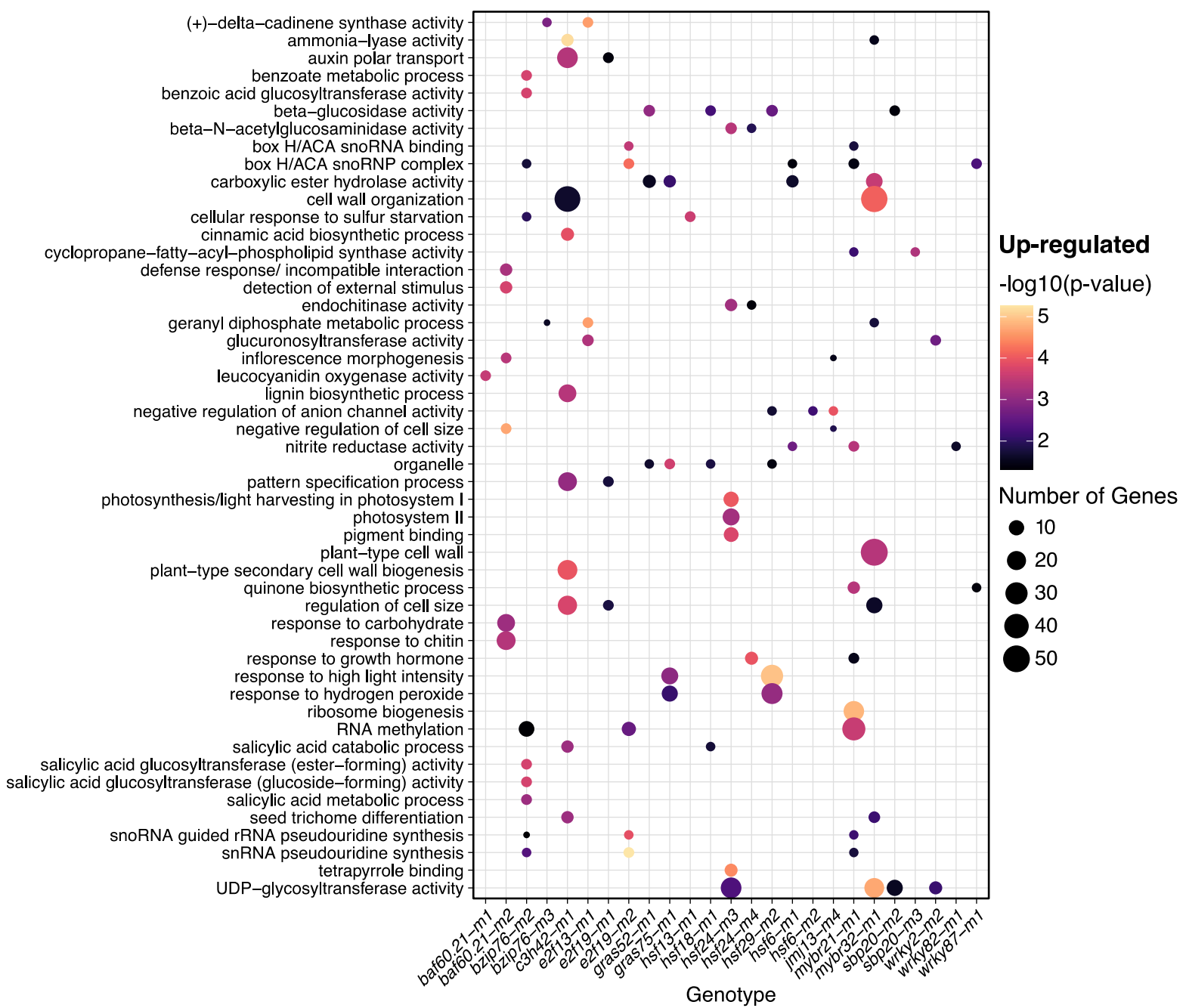

Supplement: jkae274_Supplementary_Data [file jkae274_supplementary_data.zip › Figure_S3_G3-2024-405474.pdf]

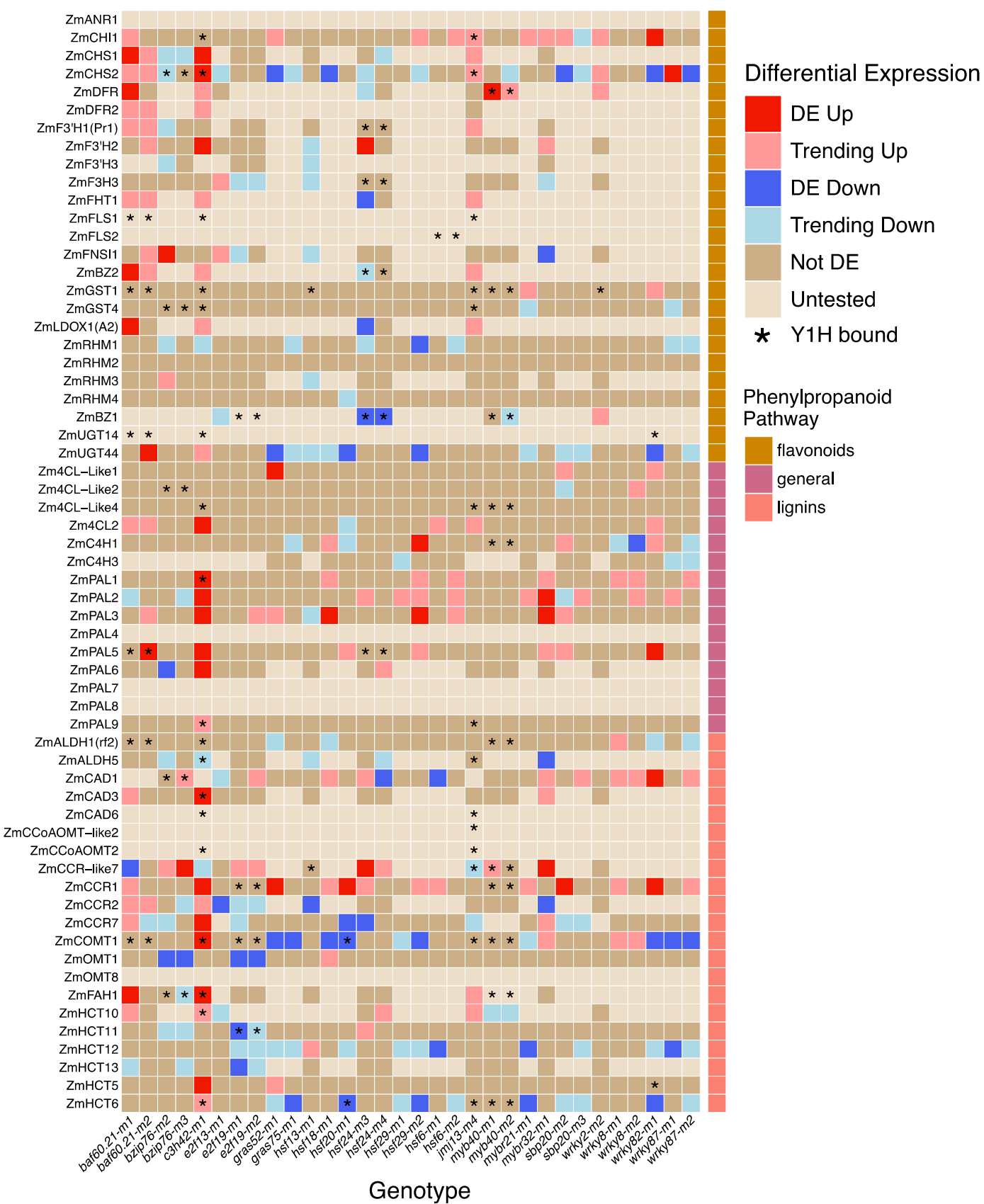

Supplement: jkae274_Supplementary_Data [file jkae274_supplementary_data.zip › Figure_S5_G3-2024-405474.pdf]

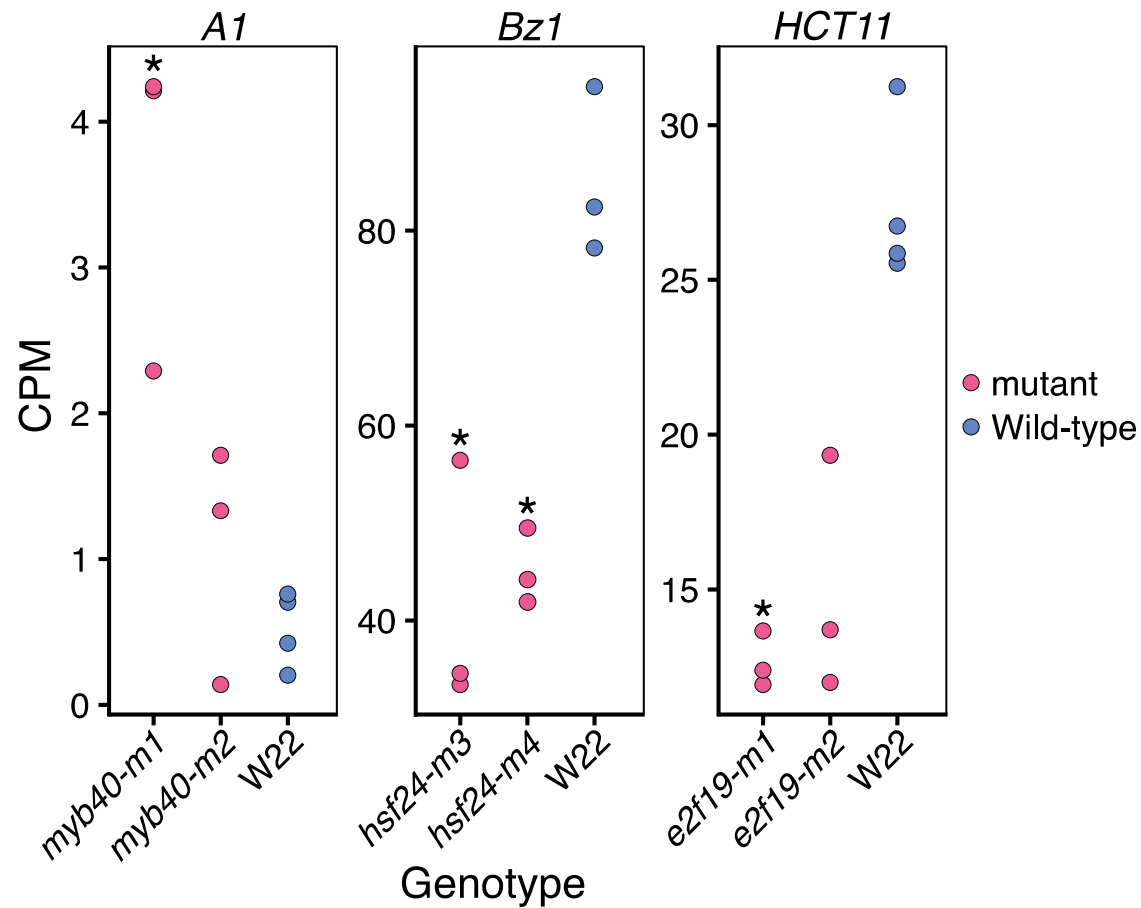

Supplement: jkae274_Supplementary_Data [file jkae274_supplementary_data.zip › Figure_S6_G3-2024-405474.pdf]

# GCN n3

**a**

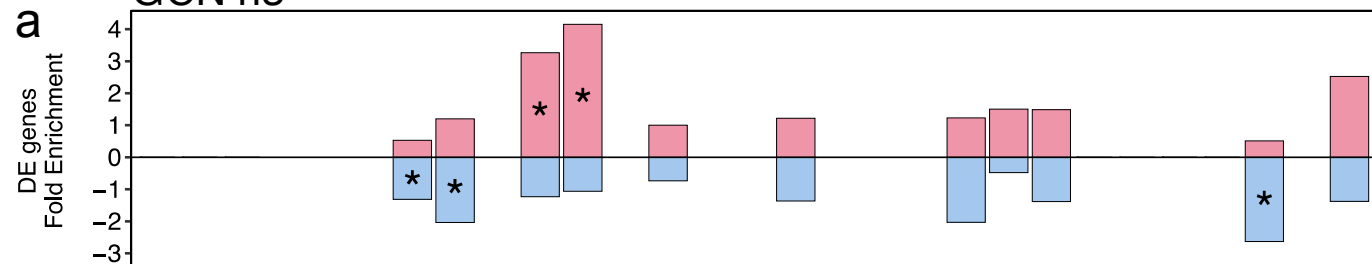

**b**

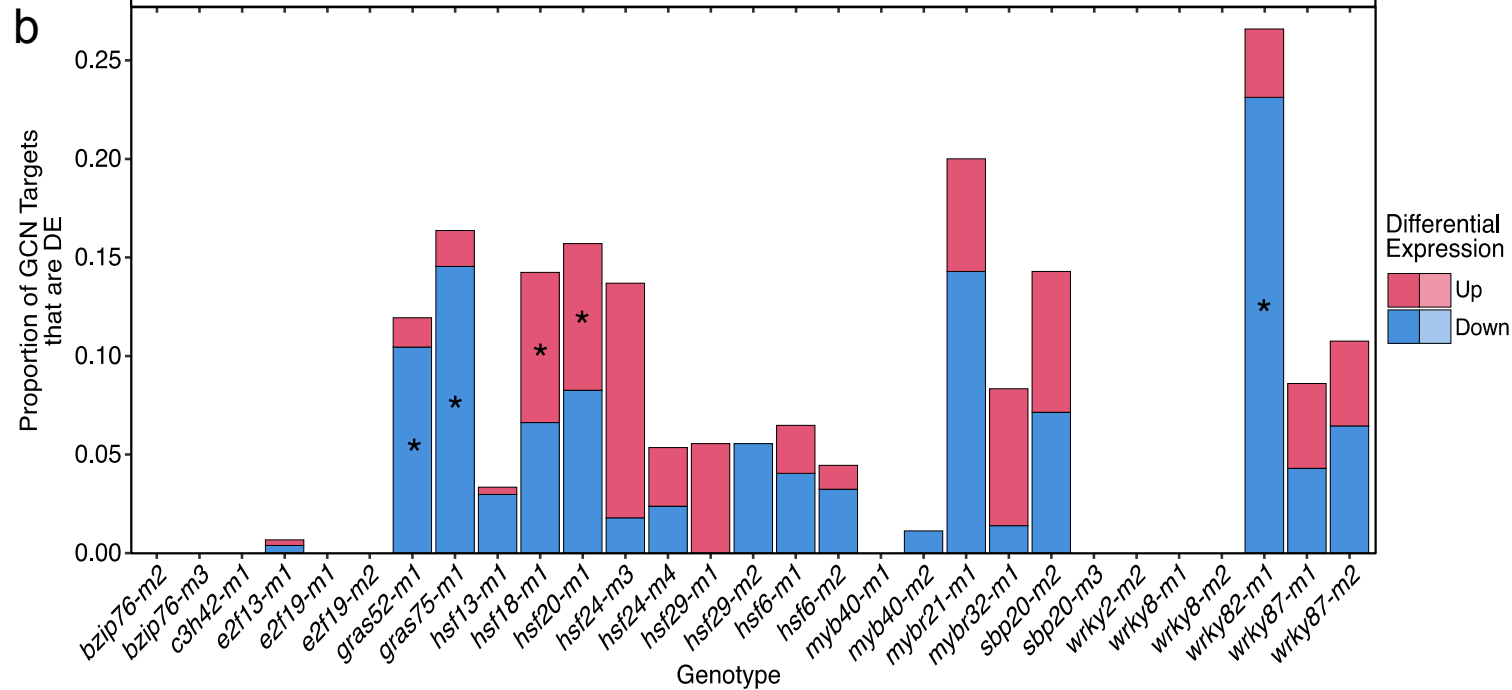

Supplement: jkae274_Supplementary_Data [file jkae274_supplementary_data.zip › Figure_S7_G3-2024-405474.pdf]
