# Supplementary material for: Transcriptome profiling of maize transcription factor mutants to probe gene regulatory network predictions
Source: G3 (Bethesda). 2024 Nov 20;15(1):jkae274. doi: 10.1093/g3journal/jkae274 (PMC11979765; doi:10.1093/g3journal/jkae274)
Supplement: jkae274_Supplementary_Data [file Figure_S4_G3-2024-405474.pdf › Figure_S4_G3-2024-405474.pdf]

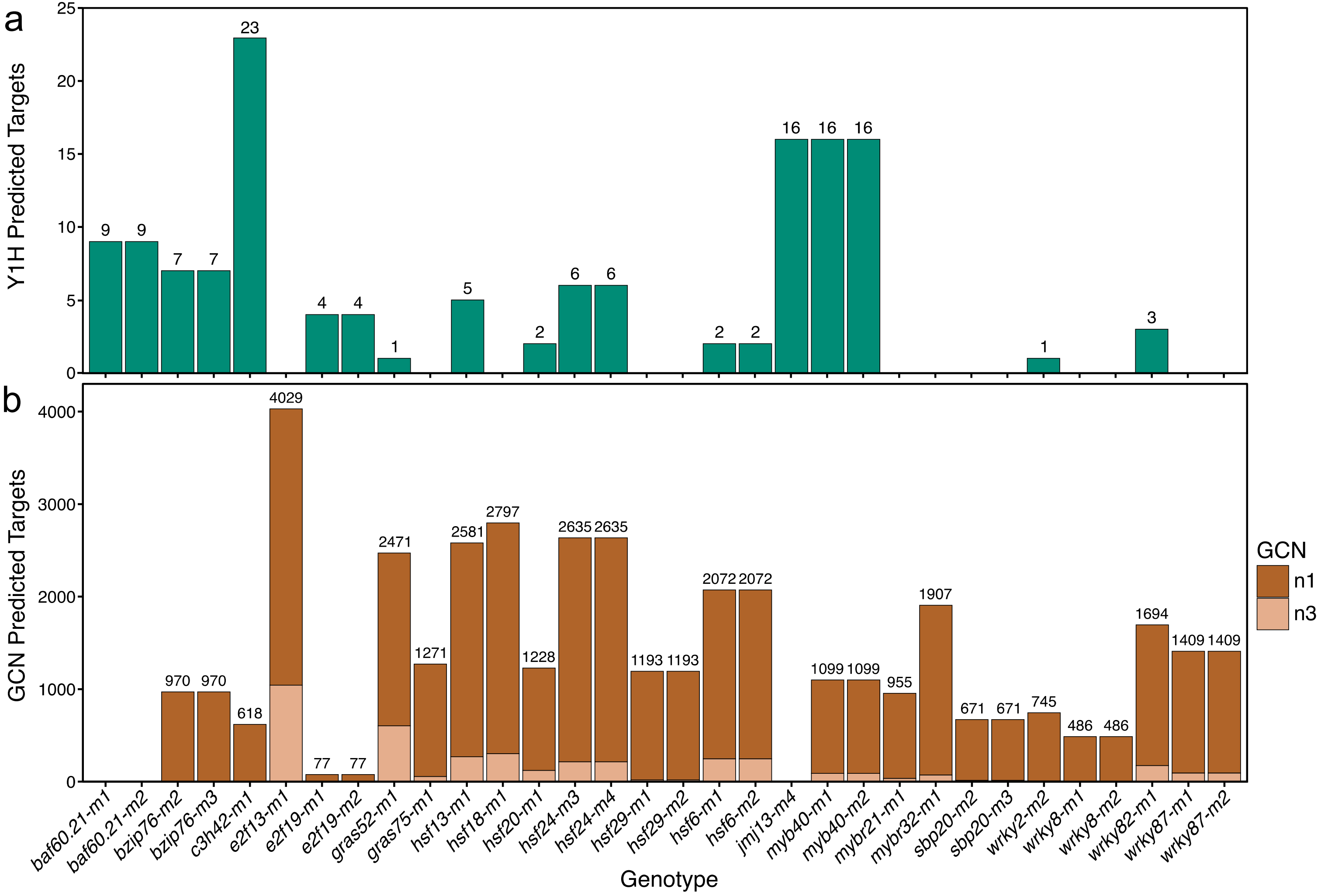
a
Y1H Predicted Targets
0
5
10
15
20
25
9
9
7
7
23
4
4
1
5
2
6
6
2
2
16
16
16
1
3
b
GCN Predicted Targets
0
1000
2000
3000
4000
970
970
618
4029
77
77
2471
1271
2581
2797
1228
2635
2635
1193
1193
2072
2072
1099
1099
955
1907
671
671
745
486
486
1694
1409
1409
GCN
n1
n3
baf60.21-m1
baf60.21-m2
bzip76-m2
bzip76-m3
c3h42-m1
e2f13-m1
e2f19-m1
e2f19-m2
gras52-m1
gras75-m1
hsf13-m1
hsf18-m1
hsf20-m1
hsf24-m3
hsf24-m4
hsf29-m1
hsf29-m2
hsf6-m1
hsf6-m2
jmj13-m4
myb40-m1
myb40-m2
mybr21-m1
mybr32-m1
sbp20-m2
sbp20-m3
wrky2-m2
wrky8-m1
wrky8-m2
wrky82-m1
wrky87-m1
wrky87-m2
Genotype
